# Kinome profiling of cholangiocarcinoma organoids reveals potential druggable targets that hold promise for treatment stratification

**DOI:** 10.1186/s10020-022-00498-1

**Published:** 2022-06-28

**Authors:** Ruby Lieshout, Alessandra V. S. Faria, Maikel P. Peppelenbosch, Luc J. W. van der Laan, Monique M. A. Verstegen, Gwenny M. Fuhler

**Affiliations:** 1 Erasmus MC Transplant Institute, Department of Surgery, Erasmus University Medical Center, Rotterdam, The Netherlands; 2 Department of Gastroenterology and Hepatology, Erasmus University Medical Center, Rotterdam, The Netherlands; 3 Department of Biochemistry and Tissue Biology, University of Campinas, UNICAMP, Campinas, Brazil

**Keywords:** Cholangiocarcinoma, Kinome profiling, Drug screening

## Abstract

**Background:**

Cholangiocarcinoma is a rare but lethal cancer of the biliary tract. Its first-line treatment is currently restricted to chemotherapy, which provides limited clinical benefit. Kinase inhibitors targeting oncogenic intracellular signaling have changed the treatment paradigm of cancer over the last decades. However, they are yet to be widely applied in cholangiocarcinoma therapy. Cholangiocarcinoma has marked molecular heterogeneity, which complicates the discovery of new treatments and requires patient stratification. Therefore, we investigated whether a commercial kinome profiling platform could predict druggable targets in cholangiocarcinoma.

**Methods:**

Kinase activity in patient-derived cholangiocarcinoma organoids, non-tumorous adjacent tissue-derived and healthy donor-derived intrahepatic cholangiocyte organoids was determined using the PamChip® phosphotyrosine kinase microarray platform. Kinome profiles were compared and correlated with RNA sequencing and (multi-)kinase inhibitor screening of the cholangiocarcinoma organoids.

**Results:**

Kinase activity profiles of individual cholangiocarcinoma organoids are different and do not cluster together. However, growth factor signaling (EGFR, PDGFRβ) and downstream effectors (MAPK pathway) are more active in cholangiocarcinoma organoids and could provide potential druggable targets. Screening of 31 kinase inhibitors revealed several promising pan-effective inhibitors and compounds that show patient-specific efficacy. Kinase inhibitor sensitivity correlated to the activity of its target kinases for several inhibitors, signifying them as potential predictors of response. Moreover, we identified correlations between drug response and kinases not directly targeted by those drugs.

**Conclusions:**

In conclusion, kinome profiling is a feasible method to identify druggable targets for cholangiocarcinoma. Future studies should confirm the potential of kinase activity profiles as biomarkers for patient stratification and precision medicine.

**Supplementary Information:**

The online version contains supplementary material available at 10.1186/s10020-022-00498-1.

## Introduction

Peptide arrays are a cost-effective, high-throughput method to determine kinase activity in cell lysates (Arsenault et al. 2011). Such kinome profiling has emerged as an effective strategy to screen activity of a large number of kinases simultaneously, and investigate how these are differentially modulated in several biological systems (Peppelenbosch et al. 2016). An advantage of this approach in cancer is that kinome profiling incorporates the complex additive effect of mutations, epigenetics, transcriptional regulation and posttranscriptional modification, resulting in a kinase activity profile that describes the downstream effect of these changes. Investigation of the global up- or downregulation of kinase activity in cancerous tissues may reveal which existing kinase inhibitors are potentially effective in patient treatment and could identify new potential druggable targets. In pediatric brain tumors, kinome profiling by peptide array confirmed previously reported signaling pathway activity in Epidermal growth factor receptor (EGFR or ERBB1), Hepatocyte growth factor receptor (c-MET) and Vascular endothelial growth factor receptor (VEGFR), associated these tumors with highly active Proto-oncogene tyrosine-protein kinase (Src) family kinases and demonstrated corresponding treatment responses to Src kinase inhibitors (Sikkema et al. 2009), showing the applicability of this technique to identify potential therapeutic targets. However, to date, kinome profiling for cholangiocarcinoma (CCA) has only been performed on 2D cell lines (Saha et al. 2016), but not primary-cancer-derived 3D organoid lines.

CCA is a relatively rare but lethal cancer originating in the biliary tract. It is the second most common form of liver cancer after hepatocellular carcinoma, representing ~ 15% of cases (Banales et al. 2020). The prognosis for CCA patients is dismal, with 5 year survival rates of 7–20% (Banales et al. 2020). CCA often goes undetected for an extended period of time, being discovered at an advanced stage when curative treatment by surgical removal has become impossible. The only systemic treatment for CCA is palliative and consists primarily of chemotherapeutics that provide limited benefit. Therefore, it is essential to identify more effective treatment modalities.

Treatments that have gained traction in recent years are the arsenal of (tyrosine) kinase inhibitors directed at molecular oncogenic intracellular signaling (Uitdehaag et al. 2019). All cellular processes depend on kinase activity, and constitutive activation of these pathways in cancerous tissues has shown to be a promising target for treatment in many cancers (Yau et al. 2015; Khan et al. 2022; Friedlaender et al. 2022). For CCA, several clinical trials were performed where such targeted therapeutics were added to palliative treatment regimens. However, results of these clinical trials with kinase inhibitors targeting Human epidermal growth factor receptor (HER), c-MET, KRAS-BRAF-MEK-ERK (Serine/threonine-protein kinase B-raf (BRAF)- Dual specificity mitogen-activated protein kinase kinase (MAP2K or MEK)-Extracellular signal-regulated kinase (ERK)), and PI3K-AKT-mTOR [Phosphoinositide 3-kinase (PI3K)- Protein kinase B (AKT)- Serine/threonine-protein kinase mTOR (mTOR)] were disappointing, as addition of the kinase inhibitors did not prove more effective than current treatment protocols (Lamarca et al. 2020). A probable explanation for the failure of these trials is that there was no accurate biological stratification of patients.

Large scale studies focused on the genetic characterization of CCA have elucidated the complex and heterogeneous mutational landscape of this tumor. These studies have found druggable mutations, amplifications or fusions of genes in about 40% of CCA patients, demonstrating notable opportunity for targeted therapies (Nakamura et al. 2015). Recently, Fibroblast growth factor receptor (FGFR) inhibitor pemigatinib was approved for treatment of CCA patients with Fibroblast growth factor receptor 2 (FGFR2) fusions or mutations, after demonstrating an objective response rate of 35.5% and a durable response of ≥ 12 months in 37% of the responders in a phase 2 clinical trial (Abou-Alfa et al. 2020). This is one example of how genetic characterization can improve treatment stratification. However, even after selection by genetic aberration, only subgroups of included patients benefit from targeted treatment. Moreover, treatment response to signaling inhibitors is not always directly linked to mutations in the targeted pathways, as multiple mutations may accumulate in a tumor, allowing it to bypass inhibitor effects (Uitdehaag et al. 2019, 2014; Tatli and Dinler 2021). Therefore, there is a pressing need for an alternative, more accurate stratification for targeted therapeutics. For CCA, correlating kinase activity to kinase inhibitor treatment response may improve personalized patient care.

As clinical trials for targeted therapies are expensive and time-consuming, a representative and scalable in vitro CCA model for drug screening could speed up the identification of personalized effective treatment approaches. With recent developments in organoid technology this has now become feasible for CCA. Patient-derived cholangiocarcinoma organoids (CCAOs) are 3-dimensional, self-organizing cell cultures that grow and recapitulate the genomic aberrations and gene expression patterns of the parental tumor to a large extent (Broutier et al. 2017; Saito et al. 2019; Maier et al. 2021; Nuciforo et al. 2018). These CCAOs can be established from both resected tumors and core needle biopsies, and are amenable to high-throughput drug screening (Broutier et al. 2017; Saito et al. 2019; Nuciforo et al. 2018).

Here, we investigated whether assessment of kinome profiles generated through a commercial platform could predict potential druggable targets in CCA. To this end, we performed phosphotyrosine kinome profiling in patient-derived CCAOs, (paired) patient-derived non-tumorous adjacent cholangiocyte organoids (HAOs), and healthy donor-derived intrahepatic cholangiocyte organoids (ICOs) to identify the differentially active kinases in CCA cells. Moreover, we screened a library of (multi-)kinase inhibitors in these CCAOs to determine if kinase activity could predict treatment response in CCA cells.

## Materials and methods

### Organoid culture

Organoids were initiated from three CCA tissue samples, two matched adjacent non-tumorous tissue samples and three healthy donor liver tissue samples. All tissue samples were collected at the Erasmus Medical Center Rotterdam after surgical resection or liver transplantation. The use of these tissue samples was approved by the medical ethics committee of Erasmus Medical Center Rotterdam (MEC-2013–143 & MEC-2014–060). All patients consented to the use of resected or transplant materials for research purposes. CCAOs and organoids from non-tumorous tissues were cultured as described previously by Broutier et al. (Broutier et al. 2017) and Huch et al. (Huch et al. 2015) CCAO1 was derived from a perihilar CCA, while CCAO2 and CCAO3 were initiated from intrahepatic CCAs. Tumorigenicity of CCAOs was confirmed by tumor formation after subcutaneous xenografting in female NOD.Cg-Prkdc^SCID^ Il2rg^tm1Wjl^/SzJ (NSG) mice (Charles River) and detection of cancer-related mutations by targeted next generation sequencing for a gene panel of 63 solid cancer-related genes (Lieshout et al. 2022, under revision). Mutations identified related to kinase signaling were *ARID1A* mutation (CCAO1) and deletion (CCAO2, CCAO3), *DDR2* amplification (CCAO2), *ERBB2* amplification (CCAO3), *FGFR1* deletion (CCAO2), *IGF1R* mutation (CCAO1) and amplification (CCAO2, CCAO3), *KRAS* mutation (CCAO1, CCAO2), *MTOR* deletion (CCAO3), *NRAS* deletion (CCAO3), *PIK3R1* deletion (CCAO2), *ROS1* deletion (CCAO2).

### Global phosphotyrosine kinase assay

#### Sample preparation

Prior to kinome profile analysis, organoids were cultured for 12 h in Advanced DMEM/F-12 (Thermo Scientific, USA) without supplements described for organoid culture, to allow full endogenous kinomic activity. After 12 h, organoids were collected and washed with NaCl 0.9% (ice cold) to remove the Matrigel. After that, the organoids were lysed using M-PER™ Mammalian Protein Extraction Reagent (Thermo Scientific, USA) with Halt Protease Inhibitor Cocktail (Thermo Scientific, USA) and Halt Protease Inhibitor Cocktail (Thermo Scientific, USA)—organoids were incubated for 10 min on ice followed by centrifugation (14,000 rpm, 10 min, 4 °C). The supernatant was collected, and protein concentration was measured using Lowry method (DC protein assay, Bio-Rad, USA). Supernatants were stored at − 80 °C until use.

#### Kinase assay

Organoid lysates (1 μg/uL protein for all samples) were loaded on a PamChip tyrosine‐kinase microarray (PamGene International BV, The Netherlands) as described previously (Baroncelli et al. 2019). Phosphorylation of the 144 kinase substrates on the array was detected by using FITC‐labelled secondary antibody. Signal intensities of the three technical replicates for each substrate were quantified using Bionavigator software (version 6.1.42.1; PamGene International BV). The internal positive control peptide ART_003_EAI(pY)AAPFAKKKXC was not considered for further analysis and the kinase activity was normalized by the CD79A_181_193 peptide. V max values below zero were artificially set to zero. Only V max values with average above zero were considered for further analysis. A dotplot graphic was built (Lux vs time 640 s to 1840s), and the area under the curve (AUC) calculated using GraphPad software (version 5.0, GraphPad Inc., USA) as a measure of peptide phosphorylation, and AUC values of zero were replaced by the lowest AUC value determined for that organoid line to allow for comparative analysis and correlation of all peptides. For upstream kinase analysis, kinases known to phosphorylate the specific peptides (target peptides) were identified by Uniprot (The 2020) and Ensembl (Howe et al. 2021) databases. The matched peptides and upstream kinases can be found in Additional file 1: Table S1.

#### Descriptive statistics and exploratory data analysis

**Fig. 1:**
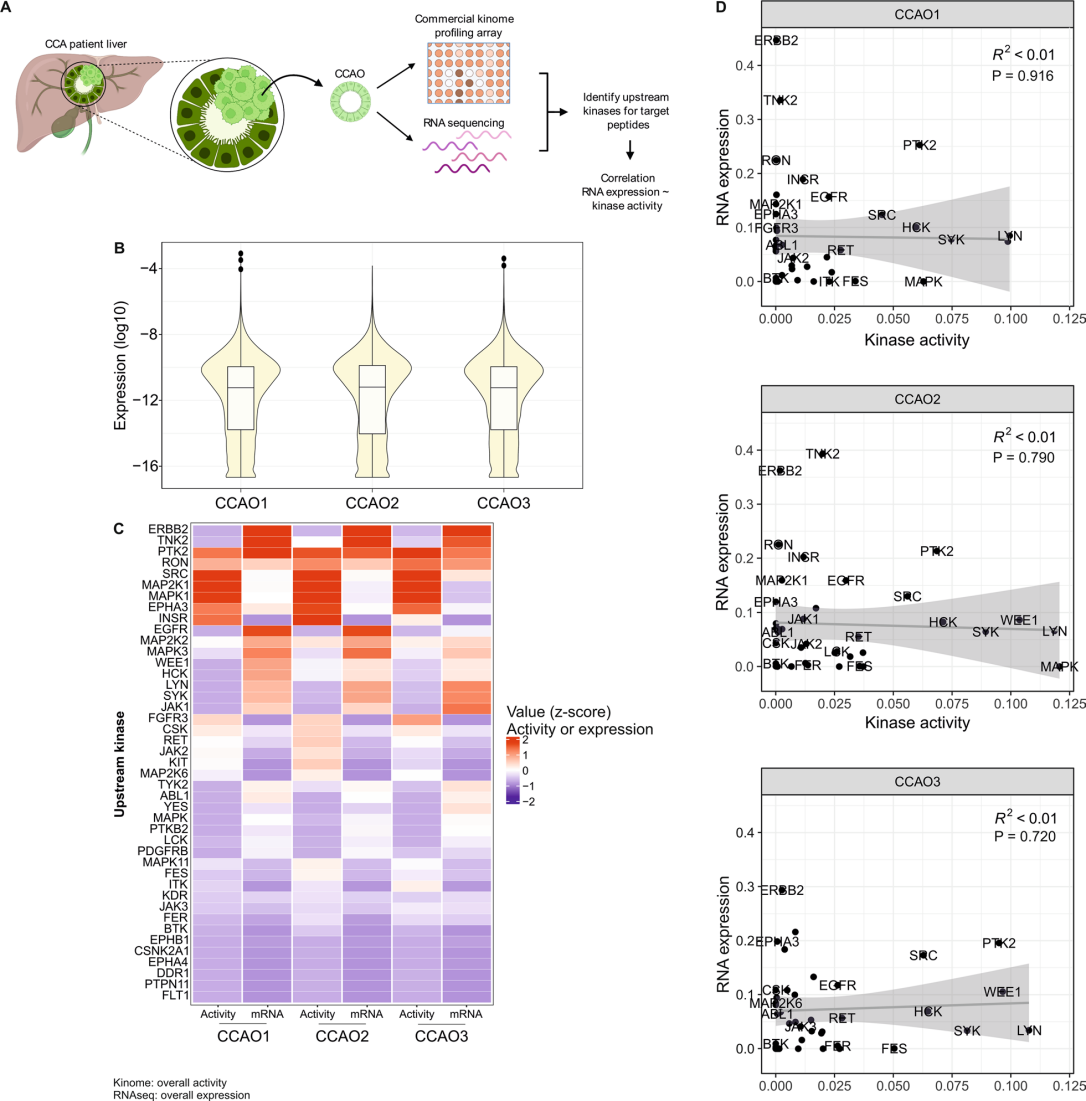
mRNA expression of kinases does not correlate to kinase activity. **A** Schematic representation of the workflow. Kinome profiles were established, and RNA sequencing data were acquired for patient-derived CCA organoids (CCAOs). Upstream kinases were identified from the Uniprot and Ensemble databases for the peptides included in the kinome profiling array. Correlation analysis was performed to investigate the relationship between mRNA expression levels and kinase activity. Schematic created with BioRender.com. **B** Violin plots with box plots of the RNA expression levels show a similar overall distribution of genes in the three CCAO lines. **C** Heatmap of the activity (AUC of the activity plots) and mRNA expression (normalized counts) of the 37 upstream kinases included in the kinome profiling array in the three CCAO lines. Data is shown by calculated z-score. **D** Scatter plots demonstrate there is no correlation between the kinase activity and RNA expression level of the upstream kinases in the three CCAOs.

**Fig. 2:**
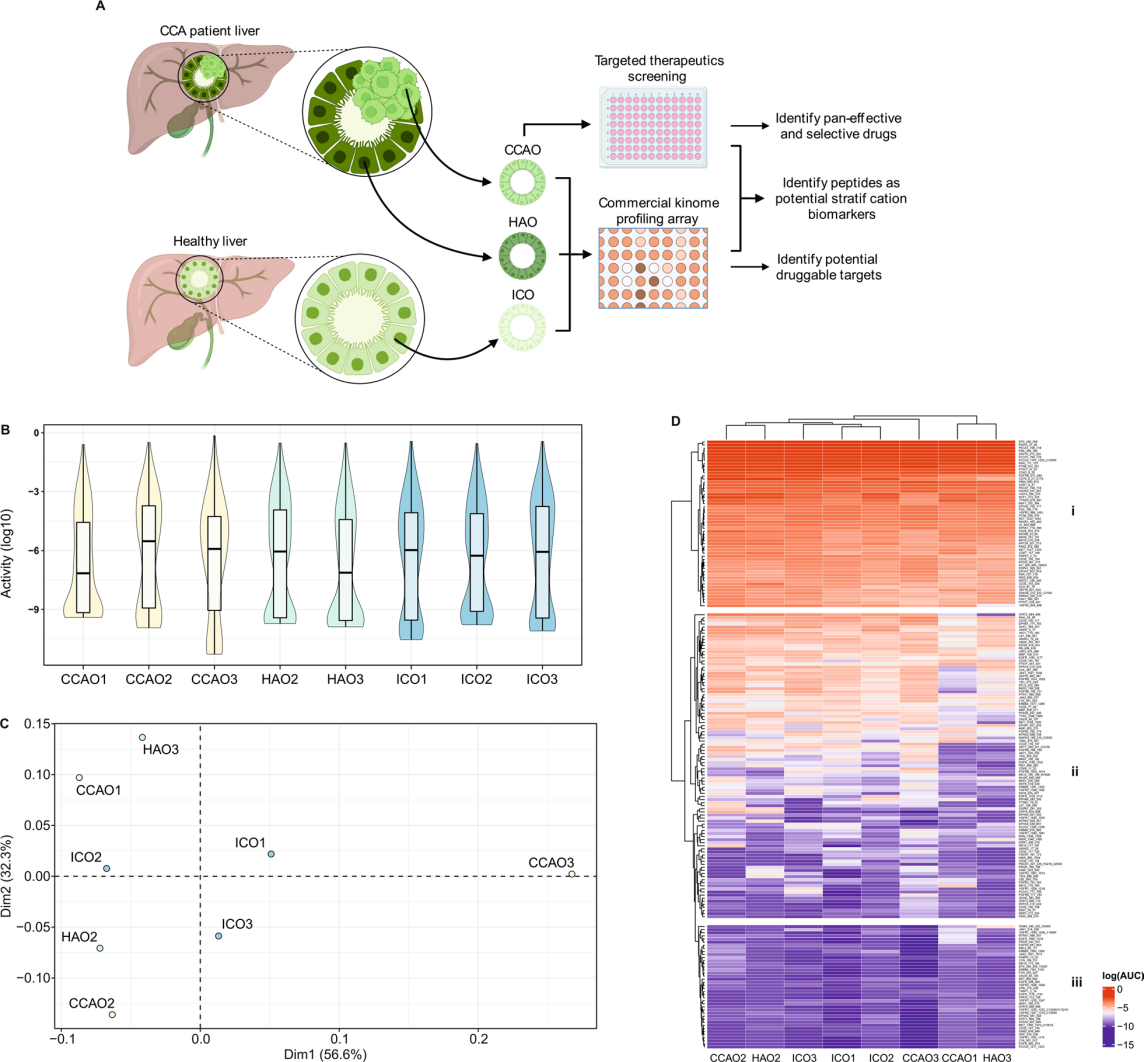
Exploratory kinome analysis demonstrates individual kinase activity profiles of CCAOs. **A** Schematic representation of the experimental setup. Kinase activity profiles were established for three CCAO lines with two matched non-tumorous adjacent tissue-derived organoid lines (CCAO1, CCAO2 with matched HAO2, and CCAO3 matched with HAO3) and three healthy donor-derived intrahepatic cholangiocyte organoid lines (ICO1, ICO2 and ICO3). Comparison of these profiles leads to the identification of potential druggable targets. CCAOs were also subjected to a screening of targeted therapeutics to identify pan-effective and selective compounds. Correlation between drug response and kinase activity leads to the discovery of potential biomarkers for treatment stratification. Schematic created with BioRender.com. **B** Violin plots with box plots showing that the overall kinase activity data density distribution is similar for all samples. Colors represent sample types (CCAO in yellow, HAO in green, ICO in blue). There was a significant difference in values among samples (p-value = 1.488e−03). **C** PCA plot of kinase activity of all peptides included in the kinome profiling array based on principle component 1 and 2. Each sample is represented by one dot (CCAO in yellow, HAO in green, ICO in blue). CCAO3 is the most distinct sample in the dataset. CCAOs are positioned far apart. **D** Heatmap of all kinase target peptides on the array analyzed by unsupervised clustering for samples and for kinase target peptides. Data is shown by log10(AUC target peptide). Three peptide clusters are emphasized: (i) peptides highly phosphorylated in all samples, (ii) peptides with heterogeneous phosphorylation patterns between samples, and (iii) peptides lowly/not phosphorylated in all samples

All exploratory data analysis were performed by R software version 4.2.1. From AUC calculated by GraphPad, data analysis was done using R 4.2.1 (Team RC 2020) and RStudio (Team R 2020) software, applying the packages tidyverse 1.3.0 (Wickham et al. 2019) for data manipulation, ggplot2 (Wickham 2016) for data visualization, factoextra 1.0.7 (Kassambara and Mundt, 2017) for Principle component analysis (PCA) analysis and graphics, complex heatmap for heatmap visualization and clustering (Gu et al. 2016), and correlation 0.7.1 (Makowski et al. 2020) for correlation calculation. Upstream kinase activity and mRNA expression heatmap (Fig. 1C) is based on calculated z-scores from AUC (kinome profiling) and Trimmed Mean of the M-values (TMM) normalized counts data (RNAseq). Clustering (Fig. 2D) is based on the distance matrix calculation (Pearson method) and followed by columns clustering.

#### Inferential statistics

All statistical analyses were performed by R software version 4.2.1. Statistical comparisons were performed by Kruskal–Wallis rank sum test (Stats R package, version 4.3.0). For correlation analysis, Spearman and Pearson methods were applied and p-values were adjusted by FDR correction. Differences were determined to be statistically significant when p < 0.05.

### RNA sequencing

#### Descriptive statistics and exploratory data analysis

GEO Dataset Record number GSE179601 based on the GeneChip Human Genome U133 Plus 2.0 Array (Affymetrix) was used to acquire RNA sequencing data of the three CCAO lines. All exploratory data analysis were performed by R software version 4.2.1. From TMM normalization data, data analysis was done using R 4.2.1 (Team RC 2020) and RStudio (Team R 2020) software, applying the packages tidyverse 1.3.0 (Wickham et al. 2019) for data manipulation, ggplot2 (Wickham 2016) for data visualization, complex heatmap for heatmap visualization and clustering (Gu et al. 2016), and correlation 0.7.1 (Makowski et al. 2020) for correlation calculation. Clustering is based on the distance matrix calculation (Pearson method) and followed by columns clustering.

#### Inferential statistics

All statistical analyses were performed by R software version 4.2.1. Statistics were made by Student’s t-test and p-values were adjusted by FDR correction. For correlation analysis, Pearson method was applied and p-values were adjusted by FDR correction. Differences were determined to be statistically significant when p < 0.05.

### FDA-approved oncology drug screening

#### Organoid treatment

Organoids were collected and washed using ice-cold Advanced DMEM, followed by mechanical disruption into fragments. Those fragments were dissociated into single cells and small cell clumps by three cycles of three minutes incubation with Trypsin–EDTA in a 37 °C water bath with mechanical disruption in between. Then, the cell suspension was filtered through a 100 µm filter, cells were plated in 5 µL droplets in white walled 96 well plates and covered with expansion medium. The Food and Drug Administration (FDA)-approved oncology drugs library version 8 used, was kindly provided by the Developmental Therapeutics Program of the Division of Cancer Treatment and Diagnosis of the National Cancer Institute (http://dtp.cancer.gov). This compound library contains 37 targeted therapeutics, of which 31 are kinase inhibitors (Additional file 1: Table S2). Compounds were added after two days of organoid culture in 1 µM and 10 µM concentrations. After four days of exposure, organoid viability was determined.

#### Viability measurement

Organoid viability was determined by quantification of ATP content using the CellTiterGlo® 3D Cell Viability Assay (Promega) according to manufacturer’s instructions. An ATP standard curve was included for every organoid line, using a 4 × dilution series ranging from 20 µM to 4.9 nM of ATP disodium salt (Promega). Drugs were considered to effectively inhibit the organoid culture if their viability value was below the mean minus three times the standard deviation of the vehicle control treated organoids of the same organoid line.

## Results

### Enzymatic kinase activities are not correlated to their transcriptional expression levels

RNA expression levels are known to correlate imperfectly to protein expression, let alone protein enzymatic activity. Therefore, we first investigated to what extent RNA transcription patterns in CCAOs are reflected in their kinomic activity (Schwanhäusser et al. 2011; Liu et al. 2016). To this end, we generated kinome profiles of three patient-derived CCAO lines to compare to established RNAseq profiles of these CCAOs (Fig. 1A). The peptide targets present on the kinome profiling array were matched to upstream kinases responsible for the phosphorylation of the target as is reported in the Uniprot and Ensemble database (Additional file 1: Table S1). Overall, similar gene expression levels were found in the three CCAO lines (CCAO1 to CCAO3) (Fig. 1B). However, as shown in Fig. 1C, mRNA expression patterns of the individual upstream kinases included in the kinome profiling array did not always correspond to activity of these enzymes. For instance, for kinases like Tyrosine-protein kinase Fes/Fps (FES) and Tyrosine-protein kinase HCK (HCK) clear activity was detected but no clear gene expression. Correlation analyses, which were performed using linear regression (Pearson method, unsupervised), confirmed that kinome profiles and RNAseq data poorly correlate in each CCAO line, as shown by R (Peppelenbosch et al. 2016) values below 0.01 (Fig. 1D). These data highlight that there is a poorly correlation between RNA expression profiles in cancer cells and the actual kinase enzymatic activity. Thus, kinome activity profiling may arguably provide a better proxy to predict kinase inhibitor response in cancer than transcriptional profiles.

### Kinome profiles are heterogeneous

We further explored the kinome profiles of the three CCAOs, and included two patient-matched non-tumorous adjacent tissue-derived cholangiocyte organoid lines (CCAO1, CCAO2 with matched HAO2, and CCAO3 matched with HAO3) and three healthy donor-derived intrahepatic cholangiocyte organoid lines (ICO1, ICO2 and ICO3) (Fig. 2A) (Marsee et al. 2021). Violin plots of the overall kinase activity levels show that activities were distributed similarly in all organoid cultures (Fig. 2B). Unsupervised cluster analysis of kinase activity in organoid samples as visualised in a principle component analysis (PCA) plot (Fig. 2C) and heatmap (Fig. 2D) demonstrated that CCAO3 expressed the most distinct kinome profile. The kinome profile of CCAO2 shows the highest similarity to its adjacent tissue counterpart HAO2, suggesting a patient-specific kinome profile for this donor (Fig. 2C, D). However, this was not observed for CCAO3 and HAO3. The healthy donor-derived ICO1 and ICO2 showed the highest similarity to each other, secondary to ICO3, demonstrating that while these lines have a diverse kinomic profile, they share more features with each other as compared to the other organoid lines tested (Fig. 2D). Aside from clustering based on samples, kinome profiles were also clustered based on peptides (Fig. 2D, Additional file 1: Table S3). Three peptide clusters were identified that embodied highly phosphorylated peptides in all samples (Fig. 2D, cluster i), lowly/not phosphorylated peptides in all samples (Fig. 2D, cluster iii), and peptides that demonstrated heterogeneous activity of kinases (Fig. 2D, cluster ii). Cluster iii peptides were excluded from further analyses as it is unlikely that kinases with very low activity could provide meaningful druggable targets. Cluster ii contains the highest number of target peptides and is likely to contain the most patient-specific information for development of CCAO-specific therapeutic targets. These findings demonstrate that there is no common kinome profile signature for CCAOs, suggesting that personalized kinase activity profiling is indeed beneficial to assess individual responses to treatment.

### Identification of shared and patient-specific therapeutic targets in CCAO

**Fig. 3:**
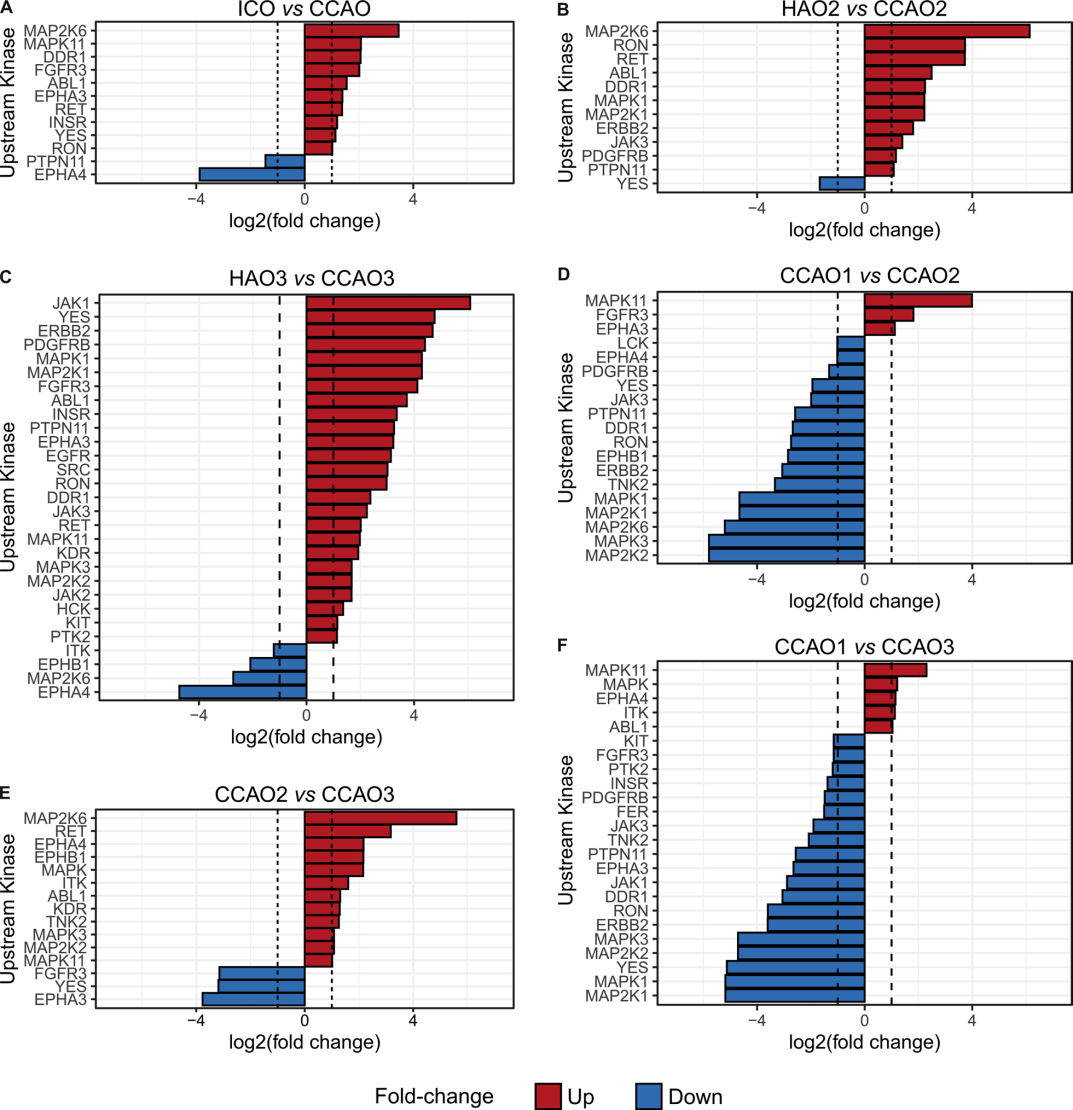
CCAOs demonstrate activity of diverse kinase pathways. Bar graphs displaying upstream kinases that have an average fold change > 2. **A** ICO (blue = higher activity) vs CCAO (red = higher activity). **B** HAO2 (blue = higher activity) vs CCAO2 (red = higher activity). **C** HAO3 (blue = higher activity) vs CCAO3 (red = higher activity). **D** CCAO2 (blue = higher activity) vs CCAO1 (red = higher activity). **E** CCAO3 (blue = higher activity) vs CCAO2 (red = higher activity). **F** CCAO3 (blue = higher activity) vs CCAO1 (red = higher activity)

We next investigated whether the CCA-derived organoids are marked by selective activity of individual kinases. To this end, we compared phosphorylation of individual peptides with known upstream kinases between the samples. Due to the low power (most comparisons are between technical triplicates of two samples to identify sample-specific patterns), no statistical analyses could be performed. Therefore, Fig. 3 displays upstream kinases responsible for phosphorylation of the array peptides with an average fold change > 2 (full dataset in Additional file 1: Table S4). The comparison between all healthy donor-derived organoids (ICO1, ICO2, ICO3) and patient-derived cholangiocarcinoma organoids CCAOs (CCAO1, CCAO2 and CCAO3) revealed 10 kinases with a fold change > 2 in CCAOs, while 2 kinases were more active in ICOs (Fig. 3A). Mitogen-activated protein kinase pathway (MAPK) family kinases (mitogen-activated protein kinase 11 (MAPK11 or p38α/SAPK2), 4.2-fold higher; and dual specificity mitogen-activated protein kinase kinase 6 (MAP2K6), 11.0-fold higher) demonstrated the highest fold change, indicating that they were more active in CCAOs (Fig. 3A). The comparison of kinase activity of matched patient samples CCAO2 and HAO2 disclosed similar findings (Fig. 3B). Eleven kinases showed at least twofold higher activity in CCAO2, while one kinase was more active in HAO2. MAPK family kinases demonstrated the largest difference in activity (MAP2K6 70.3-fold higher in CCAO2; mitogen-activated protein kinase 1 (MAPK1) and dual specificity mitogen-activated protein kinase kinase 1 (MAP2K1 or MEK1) both 4.7-fold higher in CCAO2), followed by macrophage stimulating 1 receptor (RON) (13.3-fold) and proto-oncogene tyrosine-protein kinase receptor Ret (RET) (13.2-fold) (Fig. 3B). The kinome profile of CCAO3 was very different from its adjacent counterpart, displaying 29 upstream kinases with a fold change over 2 (Fig. 3C). Janus kinase family (JAK)—(Janus kinase 1 (JAK1) 67.9-; Janus kinase 2 (JAK2) 3.2-; and Janus kinase (JAK3) 4.7-fold higher), growth factor signaling (Platelet-derived growth factor receptor beta (PDGFRβ) 23.1-fold; Fibroblast growth factor receptor 3 (FGFR3) 17.4-fold; Receptor tyrosine-protein kinase ErbB-2 (ERBB2) 25.8-fold; and EGFR 8.9-fold) and MAPK (MAP2K1 19.5-; dual specificity mitogen-activated protein kinase kinase 2 (MAP2K2 or MEK2) 3.2-; MAPK11 3.9-; and Mitogen-activated protein kinase 3 (MAPK3) 3.2-fold) kinases displayed higher activity in the cancer organoids of this patient (Fig. 3C). Comparison of the individual CCAO lines showed that the different CCAO pairs have 19, 15, and 24 upstream kinases with differential activity (fold change > 2) (Fig. 3D–F). This demonstrates that each of these CCAO lines modulates distinct kinomic pathways leading to marked diversity in kinase activity, confirming the notion that a ‘one treatment for all’ approach is not likely to be successful.

### Screening of targeted therapeutics in CCAOs reveals promising pan-effective and selective inhibitors

**Fig. 4:**
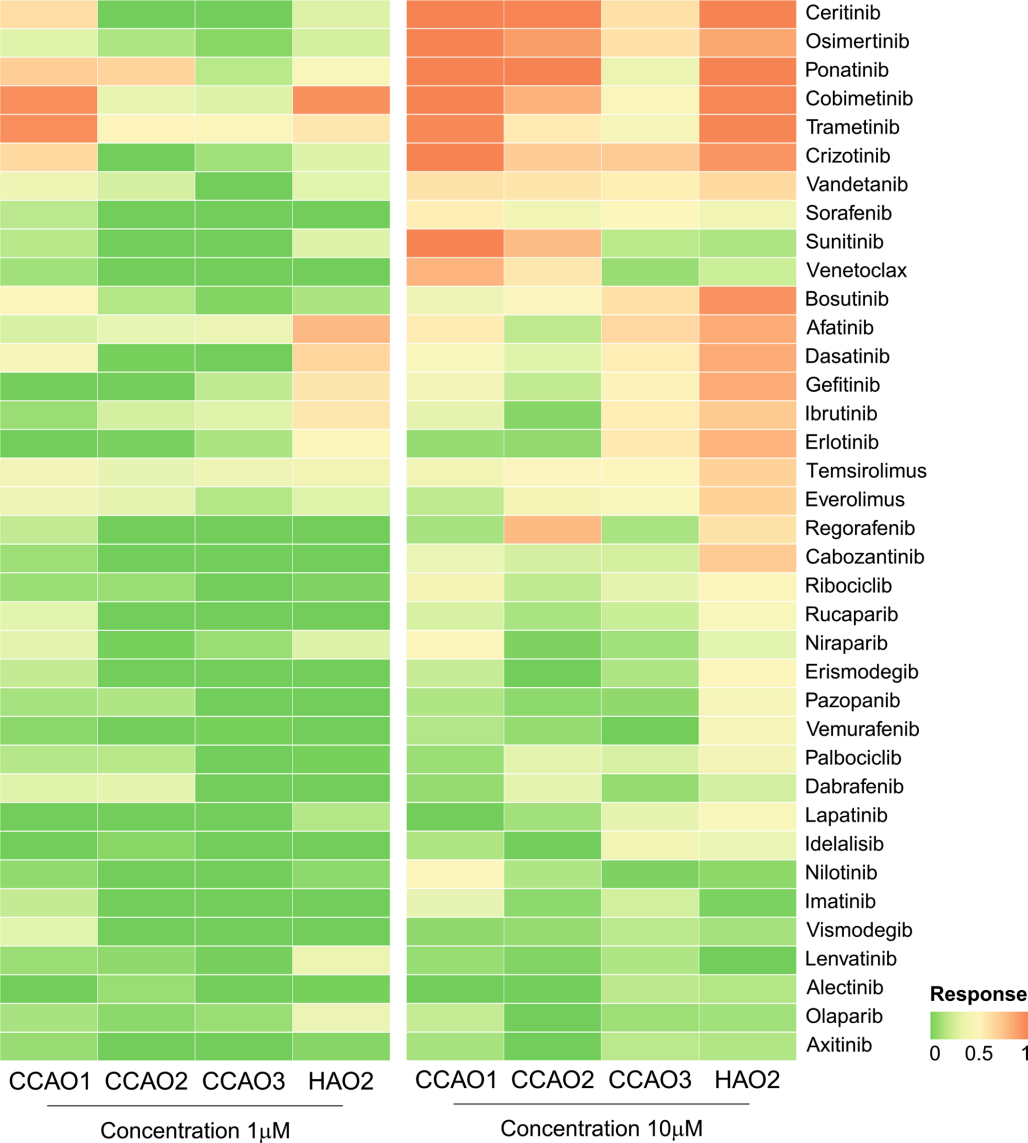
Drug screening reveals pan-effective and patient-specific drug efficacy. CCAOs and HAOs were treated with 37 FDA-approved targeted therapeutics (Additional file 1: Table S2) at 1 µM and 10 µM concentrations for four days. Drug efficacy is visualized in a heatmap of viability relative to vehicle-treated control organoids. Drug response ranges from 0, indicating that organoids are unaffected by the compound, to 1, indicating complete absence of surviving organoid cells

We investigated the sensitivity of the three CCAO lines to 37 FDA-approved targeted therapies, of which 31 are kinase inhibitors (Additional file 1: Table S2). These include multi-tyrosine kinase inhibitors (e.g. Axitinib, Sorafenib) as well as specific cytosolic kinase inhibitors (e.g. mTOR – Temsirolimus, MEK1/MEK2 – Trametinib). Organoids were subjected to treatments for four days, after which viability was determined by ATP quantification. CCAOs proved resistant to most targeted therapeutics at 1 µM (Fig. 4). Therefore, further analyses were performed with data derived from organoids treated with 10 µM of these compounds. At this concentration, eight drugs were pan-effective in CCAOs: multi-tyrosine kinase inhibitors Crizotinib, Sorafenib, Vandetanib, and Ponatinib, MEK1/MEK2 inhibitors Cobimetinib and Trametinib, EGFR inhibitor Osimertinib and Anaplastic lymphoma kinase (ALK) inhibitor Ceritinib effectively reduced viability in all three organoid lines (Fig. 4). Of these, Ponatinib and Trametinib were also effective at the lower dose of 1 μM. These pan-effective compounds could provide interesting leads for CCA treatment. In contrast, thirteen compounds demonstrated more selective drug responsiveness in one or two CCAO lines (Fig. 4). Of these, multi-tyrosine kinase inhibitor Sunitinib and B-cell lymphoma 2 (BCL-2) inhibitor Venetoclax had a selective effect for cancer organoids, inhibiting two of the CCAO lines, but leaving the third CCAO line unharmed. For these more selective inhibitors, it is important to identify stratification criteria to predict drug sensitivity. In CCAO3, activity of EGFR was higher compared to HAO3 (Fig. 3C). Interestingly, this was reflected in EGFR inhibitor sensitivity, with 5 out of 5 ErbB family of receptor tyrosine kinases (ERBB) inhibitors (Afatinib, Erlotinib, Gefitinib, Lapatinib, Osimertinib) effectively inhibiting CCAO3, while only 2/5 (CCAO1) or 1/5 (CCAO2) were effective in the other CCAO lines (Fig. 4). This promising finding encouraged a more thorough examination of the correlations between kinase activity and drug sensitivity.

### Exploring kinase activity as a drug sensitivity predictor

**Fig. 5:**
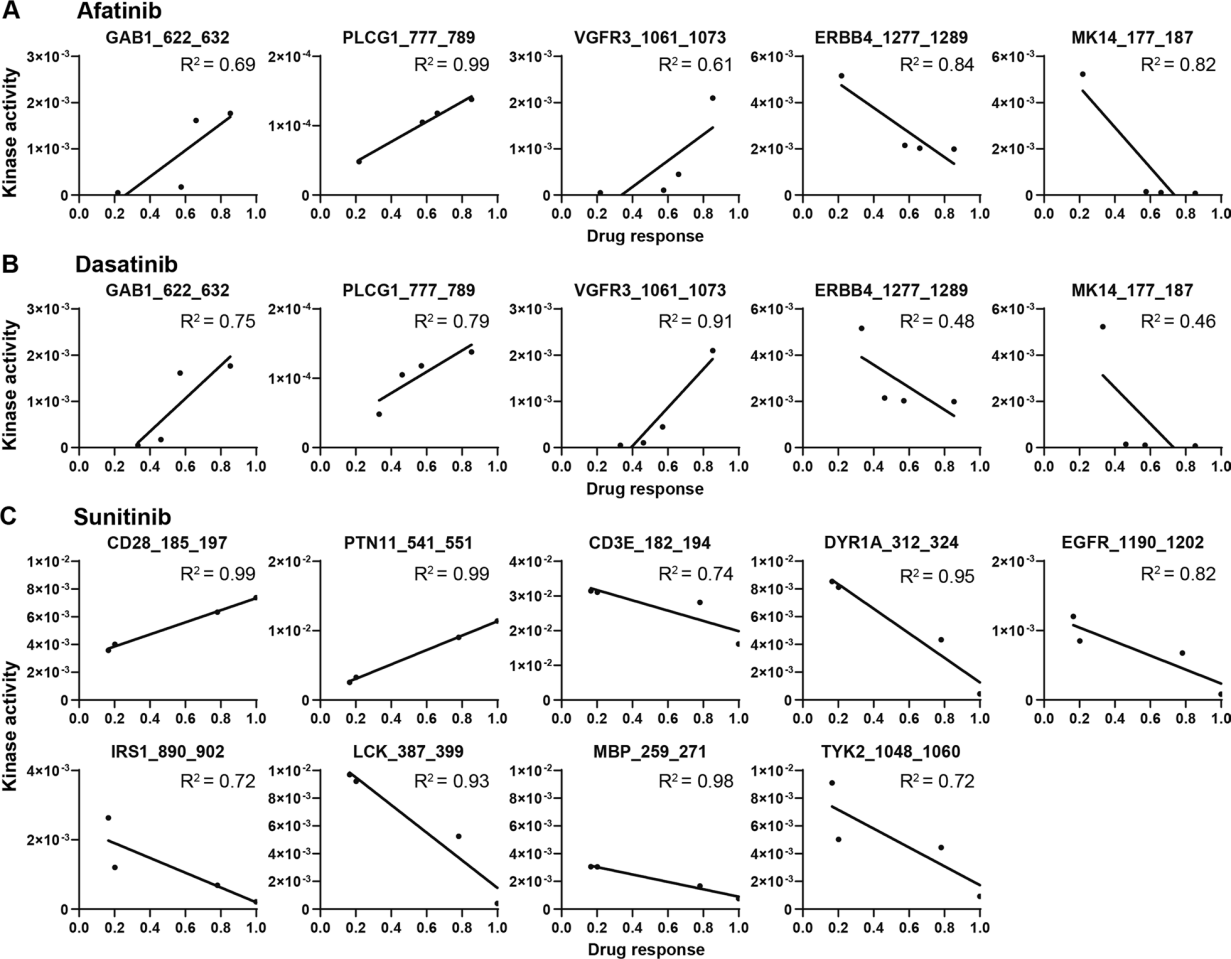
Explorative correlation analysis of drug sensitivity and kinase activity. Spearman correlation analysis revealed which array peptides correlated with Afatinib (**A**), Dasatinib (**B**) and Sunitinib (**C**) treatment sensitivity. Drug response ranges from 0, indicating that organoids are unaffected by the compound, to 1, indicating there no surviving organoid cells. Significantly correlated peptides are portrayed as scatter plots with linear regression and R^2^ values

To investigate if kinase activity could potentially provide stratification criteria for response to targeted therapeutics, we applied Spearman correlation analysis to the kinome profiling array targets and drug sensitivity data. Significantly correlated target peptides were selected for each drug, and their R^2^ values were determined by linear regression (Additional file 1: Table S5). From the compounds that showed selective effects, efficiently inhibiting some organoid lines, but not others (Fig. 4), we selected Afatinib, Dasatinib and Sunitinib to include in Fig. 5. They were chosen based on their drug target or drug response pattern (Fig. 4). Afatinib targets EGFR and serves as an example of a kinase inhibitor with a more specific target, while Dasatinib serves as an example of a multi-kinase inhibitor. Sunitinib was included because it is the drug that specifically affected tumor organoids (CCAOs) and not the non-tumorous HAOs.

Afatinib is a selective inhibitor of all members of the ERBB receptor family: EGFR, ERBB2, Receptor tyrosine-protein kinase ErbB-3 (ERBB3) and Receptor tyrosine-protein kinase ErbB-4 (ERBB4). It was effective in 2/3 CCAO lines (Fig. 4), and spearman correlation identified 5 target peptides of which phosphorylation was significantly correlated to Afatinib sensitivity (Fig. 5A). Three of these peptides displayed a positive correlation in linear regression: GAB1_622_632 (GRB2-associated-binding protein 1, GAB1), VGFR3_1061_1073 (VEGFR), and PLCG1_777_789 (1-phosphatidylinositol 4,5-bisphosphate phosphodiesterase gamma-1, PLC-γ1), and two displayed a negative correlation: ERBB4_1277_1289 (ERBB4) and MK14_177_187 (p38α/SAPK2). PLC-γ1 (Tyr 783) showed the strongest correlation with an R^2^ of 0.99. This peptide is phosphorylated by several kinases, including EGFR (Roskoski 2014), one of the receptors that is specifically inhibited by Afatinib, which could explain the correlation between Afatinib sensitivity and PLC-γ1 (Tyr 783) phosphorylation. Counter-intuitively, ERBB4 (Tyr1284) is inversely correlated to Afatinib sensitivity (Fig. 5A). The ERBB receptor family members form homo- and heterodimers that are capable of autocatalytic phosphorylation (Roskoski 2014; Kaushansky et al. 2008). Therefore, one might expect that activity of ERBB family kinases would correlate to Afatinib response, but this was not detected in this study.

Dasatinib is a multi-tyrosine kinase inhibitor known to inhibit the mutant BCR-ABL tyrosine kinase (BCR-ABL, a product from the genetic abnormality known as the Philadelphia chromosome (Ren 2005)) as its primary target, with Src family kinases (Tyrosine-protein kinase Lck (Lck), Tyrosine-protein kinase Yes (Yes), Tyrosine-protein kinase Fyn (Fyn), Src), c-KIT, and PDGFRβ as secondary targets. BCR-ABL fusions have not been described in CCA, so efficacy of Dasatinib is likely through inhibition of endogenously expressed ABL (Wang et al. 2020) or secondary target inhibition. Src family kinases were strongly inhibited in intrahepatic CCA cell lines treated with Dasatinib, indicating they are a critical target of Dasatinib in CCA (Saha et al. 2016). Drug screening in CCAOs showed that it effectively inhibits 2/3 CCAO lines (Fig. 4) and drug response correlates to the same peptides as Afatinib (Fig. 5A, B). Of these, PLCG1_777_789 (PLC-γ1) phosphorylation displayed a strong positive correlation to Dasatinib sensitivity (R^2^ = 0.79). The PLC-γ1 (Tyr 783) peptide is also a downstream target of several Src family kinases (Src, HCK, Lyn), which could provide the link between Dasatinib sensitivity and PLC-γ1 phosphorylation. The Vascular endothelial growth factor receptor 3 (VEGFR3), (Tyr1063, 1068) peptide also showed a strong positive correlation to Dasatinib (R^2^ = 0.91). This peptide is phosphorylated by VEGFR3 autocatalysis (Salameh et al. 2005). A potential relationship between Dasatinib and VEGFR3 activity could be further explored.

For Sunitinib, the only kinase inhibitor that selectively inhibits cell viability of 2/3 CCAOs without affecting HAOs, 9 peptides displayed significant correlations with drug sensitivity (Fig. 5C): CD28_185_197, CD3E_182_194, DYR1A_312_324, EGFR_1190_1202, IRS1_890_902, LCK_387_399, MBP_259_271, PTN11_541_551, TYK2_1048_1060, of which CD28_185_197 [T-cell-specific surface glycoprotein CD28 (CD28)] and PTN11_541_551 [Tyrosine-protein phosphatase non-receptor type 11 (SHP2)] showed a strong positive correlation (R^2^ = 0.99 and 1.00 respectively) (Fig. 5C). While the upstream kinase responsible for phosphorylation of CD28 (Tyr188) is unknown, making its relationship to Sunitinib sensitivity difficult to determine, the SHP2 (Tyr546/551) peptide is a target of the kinase Platelet-derived growth factor receptor alpha (PDGFRα). Sunitinib has a broad spectrum of molecular targets, including growth factor receptors PDGFRα, Platelet-derived growth factor receptor beta (PDGFRβ), Vascular endothelial growth factor receptor 1 (VEGFR1), Vascular endothelial growth factor receptor 2 (VEGFR2) and VEGFR3, tyrosine-protein kinase KIT (c-KIT), Receptor-type tyrosine-protein kinase FLT3 (FLT3), Colony stimulating factor 1 receptor (CSF1R), and proto-oncogene RET (Faivre et al. 2007). The correlation to PDGFRα-mediated phosphorylation of SHP2 (Tyr546/551) could indicate that Platelet-derived growth factor receptor (PDGFR) inhibition is potentially the most relevant mechanism through which Sunitinib affects CCAO viability. These examples demonstrate that kinase activity of both expected and unexpected kinases can correlate to drug sensitivity, which warrants further investigation as stratification markers for kinase inhibitor therapy.

## Discussion

In this study, we explored whether kinome profiles obtained through commercial platforms can predict druggable targets in CCA. First, we found that tumor-derived organoids have individual kinase activity profiles, highlighting the need for personalized treatment approaches. Second, we demonstrated that CCAO kinase activity profiles do not correlate with the RNA expression patterns of those kinases, suggesting that RNA profiles do not reflect overall kinomic outcomes. Furthermore, despite the individual patterns, CCAOs showed common increased activity of several kinases compared to their healthy counterparts, indicating these as potential druggable targets in CCAO. These include EGFR, MAPK, and Src, well-known to coordinate proliferation and progression in several cancers (Kim et al. 2009; Wagner and Nebreda 2009) Drug screening of targeted therapies revealed several pan-active compounds that show promise as CCA therapeutics. In addition, a number of kinase inhibitors showed more selective efficacy. Correlation analysis between kinase activity and drug response to these selective inhibitors demonstrated that correlations could be identified between drug sensitivity and their known drug targets for some drugs, but not all, while correlations were also found with unexpected kinases.

Kinome profiles of CCAOs were specific to the individual patients, not clustering together when compared to healthy organoid profiles. This indicates that kinase activity is highly variable between patients and highlights the need for stratification. CCA is characterized by extensive genomic heterogeneity, so it is not unexpected that this heterogeneity is also reflected in kinase activity profiles (Banales et al. 2020). Of the differentially activated kinases, MAPK kinases have been intensively studies for their role in cell proliferation, differentiation, apoptosis and migration in cancer (Wagner and Nebreda 2009). They are activated by growth factor receptor signaling (e.g. EGFR, PDGFR), cytokines, and environmental stress signals, through, amongst others, activation of the GTPase Ras. Interestingly, we found that MEK1/MEK2 inhibitors Cobimetinib and Trametinib effectively reduced organoid viability in all CCAO lines, correspondent with the overactive MAPK kinases. These findings agree with earlier preclinical studies where MEK inhibitors reduced growth and induced cell death in CCA cell lines, with KRAS mutations as a sensitizing factor (Dong et al. 2018; Wang et al. 2019). However, in both KRAS mutant and KRAS wild type CCA mouse models, MEK inhibitors were able to repress tumor growth, again suggesting that upstream activating mutations do not always predict treatment outcomes (Dong et al. 2018; Wang et al. 2019).

In line with enhanced MEK1/MEK2 signaling, EGFR and PDGFR activity were also higher in CCAOs compared to HAOs. While EGFR mutations are uncommon, occurring in ~ 5% of CCA patients, EGFR overexpression is reported regularly and has been associated with poor prognostic factors (Pellat et al. 2018). Based on our EGFR data, we have demonstrated that unbalanced EGFR signaling was an individual trait. High EGFR activity was mainly identified in CCAO3 organoids compared to its adjacent tissue counterparts. Correspondingly, CCAO3 organoids were more susceptible to ERBB (EGFR, ERBB2, ERBB3, ERBB4) family inhibitors than the other CCAOs.

A subset of targeted therapeutics displayed CCAO line-specific efficacy. An exploratory correlation analysis between kinase activity and kinase inhibitor efficacy was applied to identify potential stratification biomarkers. This led to both expected and unexpected correlations. For Afatinib, an ERBB family inhibitor, and Dasatinib, a multi-tyrosine kinase inhibitor, we found a positive correlation with PLC-γ1 (Tyr783) and VEGFR3 (Tyr1063,1068) peptides. While the upstream kinases of PLC-γ1 (Tyr783) were direct targets of these inhibitors, the relationship with VEGFR3 phosphorylation is more difficult to explain. Not every inhibitor was correlated to one of their target kinases. For example, MEK1/MEK2 inhibitor Trametinib did not correlate to MEK/ERK pathway kinase activity, and multi-kinase inhibitor Pazopanib did not correlate with its main targets c-KIT, FGFR, PDGFR, and VEGFR. There are several factors that could explain this discrepancy. First, there are several peptides on the kinome profiling array for which the upstream kinase remains unidentified, or which are phosphorylated by more than one kinase, complicating the analysis of the kinome profiles. Second, most kinase inhibitors are multi-kinase inhibitors with a broad spectrum of targets with different affinity and different efficacy per target. It is difficult to determine the impact of the activity of each kinase substrate related to an inhibitor on the kinase inhibitor sensitivity. Moreover, kinase signaling is a complex system with crosstalk between pathways and shared downstream effectors. Negative effects of inhibition of a specific kinase could be circumvented by the cancer cell via other pathways. However, every inhibitor correlated to at least one peptide. The correlations to unexpected array peptides could be identified by chance, or could potentially indicate new targets of the (multi-)kinase inhibitors, downstream effectors of the drug targets, activity of upstream kinases not yet associated with the peptide, or kinases involved via more intricate pathways. Nevertheless, mechanistic understanding of the correlation is not an absolute requirement for a kinome profiling target to become a potentially valuable biomarker for treatment stratification.

We acknowledge several limitations in our study. First, we were able to include a limited number of organoid cultures, limiting the statistical power of our analyses. Thus, we tried to avoid over-interpretation of our data by reporting fold changes rather than statistical significance levels. Second, while we included HAO2 for our drug screening analysis, normal adjacent tissue may have already been influenced by its cancerous environment. Indeed, HAO2 showed a high overlap with CCAO2 in terms of kinomic profile, suggesting that its phenotype is already affected. Drug screening further revealed that the HAOs were efficiently killed by many of the targeted therapeutics. This is perhaps unsurprising, as HAOs do not possess the genetic alterations that CCAOs do, which provides the CCAOs with resistance to certain inhibitors, but may also reflect a somewhat transformed nature of ‘normal’ cholangiocytes in cancer patients. Nevertheless, although one might worry about side effects due to damage to healthy cholangiocytes in patients treated with these inhibitors, clinical studies describe skin disease, gastrointestinal complaints and hematological disorders as common side effects, rather than biliary tree toxicity (Dungo and Keating 2013; Aparicio-Gallego et al. 2011; Wright and McCormack 2013; Wells et al. 2012). Thus, HAO death likely does not reflect patient risk.

Future studies in larger patient/control organoid cohorts which are subjected to kinome profiling and kinase inhibitor screening are needed to confirm the correlations we identified, and determine their predictive value. Some of these targets could turn out to be new biomarkers to allocate patients to specific kinase inhibitors. Furthermore, once these correlations are confirmed, future studies could attempt to discover how these kinases relate to the kinase inhibitors investigated. One approach could be to apply kinome profiling before and after treatment, to identify which kinases are affected by the compound.

In conclusion, kinome profiling demonstrated individual kinase activity patterns for each organoid culture, confirming CCA heterogeneity on a kinase activity level. EGFR, PDGFRβ, and MAPK are potential druggable targets for CCA, as they seem more active in CCAOs compared to their healthy counterparts. Drug screening identified several promising pan-effective drugs, and inhibitors that portrayed a more selective effect for specific CCAOs. Explorative correlation analysis between these compounds and kinase activity identified correlations to expected and unexpected kinase targets, which could be further investigated as potential stratification biomarkers for kinase inhibitor sensitivity.

## Supplementary Information

**Additional file 1.** Additional tables.

## Data Availability

The RNAseq datasets analyzed during the current study are available in the GEO Dataset Record repository, number GSE179601. The kinome profiling datasets used and analyzed during the current study are available from the corresponding author on reasonable request.

## Abbreviations

PLC-γ1: 1-Phosphatidylinositol 4,5-bisphosphate phosphodiesterase gamma-1
ALK: Anaplastic lymphoma kinase
A**UC**: Area under the curve
BCL-2: B-cell lymphoma 2
BCR-ABL: Bcr-Abl tyrosine-kinase
CCA: Cholangiocarcinoma;
CSF1R: Colony stimulating factor 1 receptor
NSG: NOD.Cg-Prkdc^SCID^ Il2rg^tm1Wjl^/SzJ
MAP2K or MEK: Dual specificity mitogen-activated protein kinase kinase
MAP2K1or MEK1: Dual specificity mitogen-activated protein kinase kinase 1
MAP2K2 or MEK2: Dual specificity mitogen-activated protein kinase kinase 2
MAP2K6: Dual specificity mitogen-activated protein kinase kinase 6
EGFR or ERBB1: Epidermal growth factor receptor
ERBB: ErbB family of receptor tyrosine kinases
ERK: Extracellular signal-regulated kinase
FDR: False discovery rate
FGFR: Fibroblast growth factor receptor
FGFR3: Fibroblast growth factor receptor 3
FDA: Food and Drug Administration
GAB1: GRB2-associated-binding protein 1
ICO: Healthy donor-derived intrahepatic cholangiocyte organoids
c-MET: Hepatocyte growth factor receptor
HER: Human epidermal growth factor receptor
JAK3: Janus kinase
JAK1: Janus kinase 1
JAK2: Janus kinase 2
JAK: Janus kinase family
RON: Macrophage stimulating 1 receptor
MAPK: Mitogen-activated protein kinase pathway
MAPK1: Mitogen-activated protein kinase 1
MAPK11 or p38α/SAPK2: Mitogen-activated protein kinase 11
MAPK3: Mitogen-activated protein kinase 3
CCAO: Patient-derived cholangiocarcinoma organoids
HAO: Patient-derived non-tumorous adjacent tissue-derived organoids
PI3K: Phosphoinositide 3-kinase
PDGFR: Platelet-derived growth factor receptor
PDGFRα: Platelet-derived growth factor receptor alpha
PDGFRβ: Platelet-derived growth factor receptor beta
PCA: Principle component analysis
AKT: Protein kinase B
Src: Proto-oncogene tyrosine-protein kinase
RET: Proto-oncogene tyrosine-protein kinase receptor Ret
c-KIT: Tyrosine-protein kinase KIT
ERBB2: Receptor tyrosine-protein kinase ErbB-2
ERBB3: Receptor tyrosine-protein kinase ErbB-3
ERBB4: Receptor tyrosine-protein kinase ErbB-4
FLT3: Receptor-type tyrosine-protein kinase FLT3
BRAF: Serine/threonine-protein kinase B-raf
mTOR: Serine/threonine-protein kinase mTOR
CD28: T-cell-specific surface glycoprotein CD28
TMM: Trimmed Mean of the M-values
FES: Tyrosine-protein kinase Fes/Fps
Fyn: Tyrosine-protein kinase Fyn
HCK: Tyrosine-protein kinase HCK
Lck: Tyrosine-protein kinase Lck
Yes: Tyrosine-protein kinase Yes
SHP2: Tyrosine-protein phosphatase non-receptor type 11
VEGFR: Vascular endothelial growth factor receptor
VEGFR1: Vascular endothelial growth factor receptor 1
VEGFR2: Vascular endothelial growth factor receptor 2
VEGFR3: Vascular endothelial growth factor receptor 3

## References

[CR1] Abou-Alfa GK, Sahai V, Hollebecque A, Vaccaro G, Melisi D, Al-Rajabi R, Paulson AS, Borad MJ, Gallinson D, Murphy AG, Oh DY, Dotan E (2020). Pemigatinib for previously treated, locally advanced or metastatic cholangiocarcinoma: a multicentre, open-label, phase 2 study. Lancet Oncol.

[CR2] Aparicio-Gallego G, Blanco M, Figueroa A, García-Campelo R, Valladares-Ayerbes M, Grande-Pulido E, Antón-Aparicio L (2011). New insights into molecular mechanisms of sunitinib-associated side effects. Mol Cancer Therap.

[CR3] Arsenault R, Griebel P, Napper S (2011). Peptide arrays for kinome analysis: new opportunities and remaining challenges. Proteomics.

[CR4] Banales JM, Marin JJG, Lamarca A, Rodrigues PM, Khan SA, Roberts LR, Cardinale V, Carpino G, Andersen JB, Braconi C, Calvisi DF, Perugorria MJ (2020). Cholangiocarcinoma 2020: the next horizon in mechanisms and management. Nat Rev Gastroenterol Hepatol.

[CR5] Baroncelli M, Fuhler GM, van de Peppel J, Zambuzzi WF, van Leeuwen JP, van der Eerden BCJ, Peppelenbosch MP (2019). Human mesenchymal stromal cells in adhesion to cell-derived extracellular matrix and titanium: Comparative kinome profile analysis. J Cell Physiol.

[CR6] Broutier L, Mastrogiovanni G, Verstegen MM, Francies HE, Gavarro LM, Bradshaw CR, Allen GE, Arnes-Benito R, Sidorova O, Gaspersz MP, Georgakopoulos N, Koo BK (2017). Human primary liver cancer-derived organoid cultures for disease modeling and drug screening. Nat Med.

[CR7] Dong M, Liu X, Evert K, Utpatel K, Peters M, Zhang S, Xu Z, Che L, Cigliano A, Ribback S, Dombrowski F, Cossu A (2018). Efficacy of MEK inhibition in a K-Ras-driven cholangiocarcinoma preclinical model. Cell Death Dis.

[CR8] Dungo RT, Keating GM (2013). Afatinib: first global approval. Drugs.

[CR9] Faivre S, Demetri G, Sargent W, Raymond E (2007). Molecular basis for sunitinib efficacy and future clinical development. Nat Rev Drug Discov.

[CR10] Friedlaender A, Subbiah V, Russo A, Banna GL, Malapelle U, Rolfo C, Addeo A (2022). EGFR and HER2 exon 20 insertions in solid tumours: from biology to treatment. Nat Rev Clin Oncol.

[CR11] Gu Z, Eils R, Schlesner M (2016). Complex heatmaps reveal patterns and correlations in multidimensional genomic data. Bioinformatics.

[CR12] Howe KL, Achuthan P, Allen J, Allen J, Alvarez-Jarreta J, Amode MR, Armean IM, Azov AG, Bennett R, Bhai J, Billis K, Boddu S (2021). Ensembl 2021. Nucleic Acids Res.

[CR13] Huch M, Gehart H, van Boxtel R, Hamer K, Blokzijl F, Verstegen MM, Ellis E, van Wenum M, Fuchs SA, de Ligt J, van de Wetering M, Sasaki N (2015). Long-term culture of genome-stable bipotent stem cells from adult human liver. Cell.

[CR14] Kassambara A, Mundt F. Package ‘factoextra’. Extract and visualize the results of multivariate data analyses. 2017. https://CRAN.R-project.org/package=factoextra.

[CR15] Kaushansky A, Gordus A, Budnik BA, Lane WS, Rush J, MacBeath G (2008). System-wide investigation of ErbB4 reveals 19 sites of Tyr phosphorylation that are unusually selective in their recruitment properties. Chem Biol.

[CR16] Khan PS, Rajesh P, Rajendra P, Chaskar MG, Rohidas A, Jaiprakash S (2022). Recent advances in B-RAF inhibitors as anticancer agents. Bioorg Chem.

[CR17] Kim LC, Song L, Haura EB (2009). Src kinases as therapeutic targets for cancer. Nat Rev Clin Oncol.

[CR18] Lamarca A, Barriuso J, McNamara MG, Valle JW (2020). Molecular targeted therapies: Ready for "prime time" in biliary tract cancer. J Hepatol.

[CR19] Liu Y, Beyer A, Aebersold R (2016). On the dependency of cellular protein levels on mRNA abundance. Cell.

[CR20] Maier CF, Zhu L, Nanduri LK, Kuhn D, Kochall S, Thepkaysone ML, William D, Grutzmann K, Klink B, Betge J, Weitz J, Rahbari NN (2021). Patient-derived organoids of cholangiocarcinoma. Int J Mol Sci.

[CR21] Makowski D, Ben-Shachar MS, Patil I, Lüdecke D (2020). Methods and algorithms for correlation analysis in R. JOSS.

[CR22] Marsee A, Roos FJM, Verstegen MMA, Marsee A, Roos F, Verstegen M, Clevers H, Vallier L, Takebe T, Huch M, Peng WC, Forbes S (2021). Building consensus on definition and nomenclature of hepatic, pancreatic, and biliary organoids. Cell Stem Cell.

[CR23] Nakamura H, Arai Y, Totoki Y, Shirota T, Elzawahry A, Kato M, Hama N, Hosoda F, Urushidate T, Ohashi S, Hiraoka N, Ojima H (2015). Genomic spectra of biliary tract cancer. Nat Genet.

[CR24] Nuciforo S, Fofana I, Matter MS, Blumer T, Calabrese D, Boldanova T, Piscuoglio S, Wieland S, Ringnalda F, Schwank G, Terracciano LM, Ng CKY (2018). Organoid models of human liver cancers derived from tumor needle biopsies. Cell Rep.

[CR25] Pellat A, Vaquero J, Fouassier L (2018). Role of ErbB/HER family of receptor tyrosine kinases in cholangiocyte biology. Hepatology.

[CR26] Peppelenbosch MP, Frijns N, Fuhler G (2016). Systems medicine approaches for peptide array-based protein kinase profiling: progress and prospects. Expert Rev Proteomics.

[CR27] Ren R (2005). Mechanisms of BCR-ABL in the pathogenesis of chronic myelogenous leukaemia. Nat Rev Cancer.

[CR28] Roskoski R (2014). The ErbB/HER family of protein-tyrosine kinases and cancer. Pharmacol Res.

[CR29] Saha SK, Gordan JD, Kleinstiver BP, Vu P, Najem MS, Yeo J-C, Shi L, Kato Y, Levin RS, Webber JT (2016). Isocitrate dehydrogenase mutations confer dasatinib hypersensitivity and SRC dependence in intrahepatic cholangiocarcinoma. Cancer Discov.

[CR30] Saha SK, Gordan JD, Kleinstiver BP, Vu P, Najem MS, Yeo JC, Shi L, Kato Y, Levin RS, Webber JT, Damon LJ, Egan RK (2016). Isocitrate Dehydrogenase mutations confer dasatinib hypersensitivity and src dependence in intrahepatic cholangiocarcinoma. Cancer Discov.

[CR31] Saito Y, Muramatsu T, Kanai Y, Ojima H, Sukeda A, Hiraoka N, Arai E, Sugiyama Y, Matsuzaki J, Uchida R, Yoshikawa N, Furukawa R (2019). Establishment of patient-derived organoids and drug screening for biliary tract carcinoma. Cell Rep.

[CR32] Salameh A, Galvagni F, Bardelli M, Bussolino F, Oliviero S (2005). Direct recruitment of CRK and GRB2 to VEGFR-3 induces proliferation, migration, and survival of endothelial cells through the activation of ERK, AKT, and JNK pathways. Blood.

[CR33] Schwanhäusser B, Busse D, Li N, Dittmar G, Schuchhardt J, Wolf J, Chen W, Selbach M (2011). Global quantification of mammalian gene expression control. Nature.

[CR34] Sikkema AH, Diks SH, den Dunnen WF, ter Elst A, Scherpen FJ, Hoving EW, Ruijtenbeek R, Boender PJ, de Wijn R, Kamps WA, Peppelenbosch MP, de Bont ES (2009). Kinome profiling in pediatric brain tumors as a new approach for target discovery. Cancer Res.

[CR35] Tatli O, Dinler DG (2021). Recent developments in targeting RAS downstream effectors for RAS-driven cancer therapy. Molecules.

[CR36] Team R (2020). RStudio: integrated development for R.

[CR37] Team RC (2020). A language and environment for statistical computing.

[CR38] The UC (2020). UniProt: the universal protein knowledgebase in 2021. Nucleic Acids Res.

[CR39] Uitdehaag JC, de Roos JA, van Doornmalen AM, Prinsen MB, de Man J, Tanizawa Y, Kawase Y, Yoshino K, Buijsman RC, Zaman GJ (2014). Comparison of the cancer gene targeting and biochemical selectivities of all targeted kinase inhibitors approved for clinical use. PLoS ONE.

[CR40] Uitdehaag JCM, Kooijman JJ, de Roos J, Prinsen MBW, Dylus J, Willemsen-Seegers N, Kawase Y, Sawa M, de Man J, van Gerwen SJC, Buijsman RC, Zaman GJR (2019). Combined cellular and biochemical profiling to identify predictive drug response biomarkers for kinase inhibitors approved for clinical use between 2013 and 2017. Mol Cancer Ther.

[CR41] Wagner EF, Nebreda AR (2009). Signal integration by JNK and p38 MAPK pathways in cancer development. Nat Rev Cancer.

[CR42] Wang P, Song X, Utpatel K, Shang R, Yang YM, Xu M, Zhang J, Che L, Gordan J, Cigliano A, Seki E, Evert M (2019). MEK inhibition suppresses K-Ras wild-type cholangiocarcinoma in vitro and in vivo via inhibiting cell proliferation and modulating tumor microenvironment. Cell Death Dis.

[CR43] Wang F, Hou W, Chitsike L, Xu Y, Bettler C, Perera A, Bank T, Cotler SJ, Dhanarajan A, Denning MF, Ding X, Breslin P (2020). ABL1, overexpressed in hepatocellular carcinomas, regulates expression of NOTCH1 and promotes development of liver tumors in mice. Gastroenterology.

[CR44] Wells SA, Robinson BG, Gagel RF, Dralle H, Fagin JA, Santoro M, Baudin E, Elisei R, Jarzab B, Vasselli JR (2012). Vandetanib in patients with locally advanced or metastatic medullary thyroid cancer: a randomized, double-blind phase III trial. J Clin Oncol.

[CR45] Wickham H (2016). ggplot2: elegant graphics for data analyised.

[CR46] Wickham H, Averick M, Bryan J, Chang W, McGowan LDA, François R, Grolemund G, Hayes A, Henry L, Hester J (2019). Welcome to the Tidyverse. JOSS.

[CR47] Wright CJM, McCormack PL (2013). Trametinib: first global approval. Drugs.

[CR48] Yau NK, Fong AY, Leung HF, Verhoeft KR, Lim QY, Lam WY, Wong IC, Lui VW (2015). A pan-cancer review of ALK mutations: implications for carcinogenesis and therapy. Curr Cancer Drug Targets.

